# Substantial Metabolic Activity of Human Brown Adipose Tissue during Warm Conditions and Cold-Induced Lipolysis of Local Triglycerides

**DOI:** 10.1016/j.cmet.2018.04.020

**Published:** 2018-06-05

**Authors:** Graeme Weir, Lynne E. Ramage, Murat Akyol, Jonathan K. Rhodes, Catriona J. Kyle, Alison M. Fletcher, Thomas H. Craven, Sonia J. Wakelin, Amanda J. Drake, Maria-Lena Gregoriades, Ceri Ashton, Nick Weir, Edwin J.R. van Beek, Fredrik Karpe, Brian R. Walker, Roland H. Stimson

**Affiliations:** 1 BHF/University Centre for Cardiovascular Science, University of Edinburgh, The Queen’s Medical Research Institute, 47 Little France Crescent, Edinburgh EH16 4TJ, Scotland, UK; 2 Department of Radiology, Royal Infirmary of Edinburgh, Edinburgh, Scotland, UK; 3 Department of Surgery, Royal Infirmary of Edinburgh, Edinburgh, Scotland, UK; 4 Department of Anaesthesia and Critical Care, University of Edinburgh, Edinburgh, Scotland, UK; 5 Edinburgh Imaging Facility QMRI, University of Edinburgh, Edinburgh, Scotland, UK; 6 Department of Radiology, Western General Hospital, Edinburgh, Scotland, UK; 7 Department of Medical Physics, Royal Infirmary of Edinburgh, Edinburgh, Scotland, UK; 8 Oxford Centre for Diabetes, Endocrinology and Metabolism, University of Oxford, Oxford, UK; 9 NIHR Oxford Biomedical Research Centre, OUH Trust, Oxford, UK; 10 Institute of Genetic Medicine, Newcastle University, Newcastle upon Tyne, UK

**Keywords:** brown adipose tissue, cold exposure, human, PET/CT, microdialysis, tracer, ^133^xenon, substrate utilization, physiology

## Abstract

Current understanding of *in vivo* human brown adipose tissue (BAT) physiology is limited by a reliance on positron emission tomography (PET)/computed tomography (CT) scanning, which has measured exogenous glucose and fatty acid uptake but not quantified endogenous substrate utilization by BAT. Six lean, healthy men underwent ^18^fluorodeoxyglucose-PET/CT scanning to localize BAT so microdialysis catheters could be inserted in supraclavicular BAT under CT guidance and in abdominal subcutaneous white adipose tissue (WAT). Arterial and dialysate samples were collected during warm (∼25°C) and cold exposure (∼17°C), and blood flow was measured by ^133^xenon washout. During warm conditions, there was increased glucose uptake and lactate release and decreased glycerol release by BAT compared with WAT. Cold exposure increased blood flow, glycerol release, and glucose and glutamate uptake only by BAT. This novel use of microdialysis reveals that human BAT is metabolically active during warm conditions. BAT activation substantially increases local lipolysis but also utilization of other substrates such as glutamate.

## Highlights

• Human BAT shows substantial glucose uptake and lactate release during warm conditions
• Glycerol recycling occurs in human BAT, and cold exposure activates BAT lipolysis
• BAT uses substrates other than just glucose or fatty acids during cold activation
• Microdialysis is a novel technique to measure *in vivo* human BAT physiology

## Graphical Abstract

**Figure:**
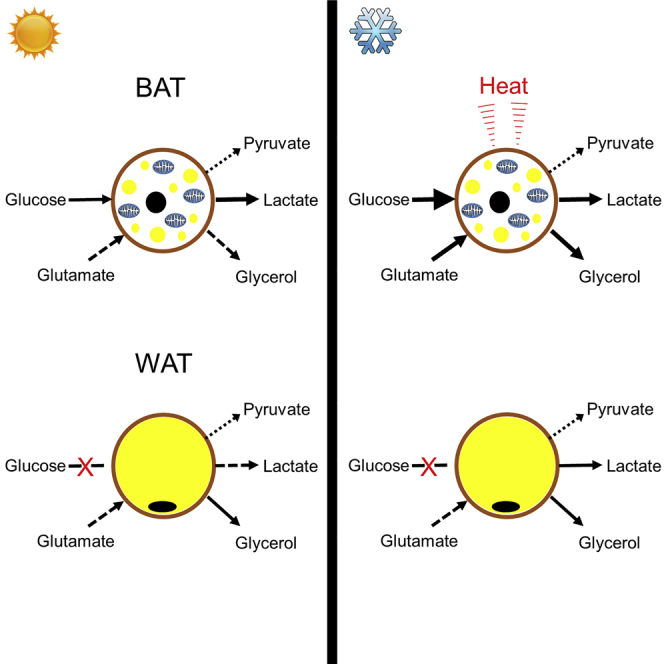

## Teaser

Weir et al., using microdialysis, have shown that human brown adipose tissue (BAT) is metabolically active in warm conditions, with higher glucose uptake and lactate release than white AT. Cold activation increased glucose and glutamate uptake and glycerol release by BAT, quantifying substrate utilization and hydrolysis of BAT triglycerides during thermogenesis.

## Introduction

The identification of brown adipose tissue (BAT) in adult humans offers the possibility of activating this tissue to treat metabolic disease. However, our understanding of *in vivo* human BAT physiology has been limited by the requirement for positron emission tomography (PET)/computed tomography (CT) to quantify BAT activity. For example, PET/CT (most commonly using ^18^fluorodeoxyglucose [^18^FDG]) has been relied upon to infer that BAT is activated by cold (Saito et al., 2009, Virtanen et al., 2009), is under sympathetic regulation (Cypess et al., 2015), contributes to non-shivering thermogenesis (Ouellet et al., 2012), enhances insulin sensitivity (Lee et al., 2014), and regulates lipid metabolism (Chondronikola et al., 2016). While these findings show that human and rodent BAT share substantial similarities, there are also important differences in the regulation of BAT activation (Ramage et al., 2016), highlighting the need to dissect BAT physiology in humans. In addition, although fatty acids are the primary substrate for BAT thermogenesis in rodents (Ma and Foster, 1986), most human studies using PET have used glucose uptake as the primary measurement of BAT activation.

Many important questions regarding substrate utilization by human BAT have not been answered using PET. For example, while there has been much attention on cold- and drug-induced BAT activation, it is unclear whether there is substantial metabolic activity by BAT during warm conditions when thermogenesis is not required. In addition, the fate of the substantial cold-induced glucose uptake by BAT is unclear, such as whether this is oxidized to generate heat or predominantly released as intermediates such as lactate and pyruvate, as in rodents (Ma and Foster, 1986). PET cannot measure the hydrolysis of local triglycerides which are believed to be the primary substrate for BAT thermogenesis (Blondin et al., 2017), and is limited by the capacity to measure uptake of one substrate at a time. This has led to a focus on glucose with limited information available on other substrate utilization by human BAT. Alternative *in vivo* techniques used to study human BAT, such as measurement of supraclavicular temperature (Lee et al., 2011, Ramage et al., 2016), MRI (Hu et al., 2013), and magnetic resonance spectroscopy (Raiko et al., 2015), have also not provided answers to these questions.

Microdialysis is a technique where a catheter with a semi-permeable membrane is inserted into a tissue of interest, an isotonic perfusate is then slowly infused through the catheter to allow the perfusate to equilibrate with the interstitial concentrations (Barbe et al., 2001), and dialysate is collected to measure compounds of interest. We adapted microdialysis, in combination with arterial and blood flow measurements and stable isotope glucose and glycerol tracer infusion, as a novel *in vivo* technique to quantify endogenous substrate utilization by human BAT and white adipose tissue (WAT) during warm conditions and during cold activation.

## Results

### Study Visit 1: Identification and Quantification of ^18^FDG Uptake by BAT

**Figure 1:**
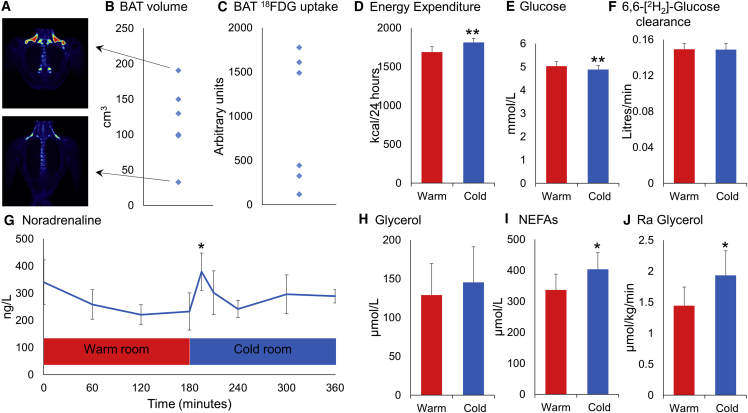
PET/CT Identification of Supraclavicular BAT and Whole-Body Measurements (A) ^18^FDG PET/CT images from subjects with the most (top panel) and least (bottom panel) BAT volume during cold exposure. (B and C) Volume of active BAT (B) and total ^18^FDG uptake by BAT (C) in each subject. (D–J) Data are mean ± SEM for (D) n = 6 and (E–J) n = 5 subjects during warm (red columns) and cold (blue columns) exposure. (D) Cold exposure (3 hr duration) increased whole-body energy expenditure in all subjects. Cold exposure (E) reduced arterial glucose but (F) did not alter the clearance of 6,6-[^2^H]_2_-glucose. Cold exposure (G) transiently increased noradrenaline concentrations, (H) did not alter glycerol but increased (I) non-esterified fatty acids (NEFAs) and (J) the rate of appearance (Ra) of glycerol. Data were analyzed using the paired t test or by repeated measures ANOVA with post hoc least significant difference (LSD) testing. ^∗^p < 0.05, ^∗∗^p < 0.01 versus warm room.

Six lean healthy men (subject characteristics in Table S1) underwent ^18^FDG-PET/CT scanning following cold exposure to quantify their active BAT (Figure S1). All six subjects had detectable ^18^FDG uptake by BAT (Figures 1A–1C), with a mean BAT volume of 117 ± 22 cm^3^. In particular, there was substantial ^18^FDG uptake by supraclavicular BAT in all subjects, which ensured they could proceed to study visit 2.

### Study Visit 2: Measurement of BAT Activity Using Microdialysis

Microdialysis catheters were placed in supraclavicular BAT and abdominal subcutaneous WAT (Figure S1). ^133^Xenon was injected into BAT and WAT to measure blood flow continuously (Astrup et al., 1985). A catheter was inserted into a radial artery for repeated sampling. Subjects were kept in a warm room (∼25°C) and then a cold room (∼17°C) each for 3 hr.

#### Whole-Body Measurements

Whole-body energy expenditure was increased by 125 ± 32 kcal/24 hr by cold exposure (Figure 1D) (p < 0.01). Cold exposure decreased peripheral skin temperature, increased systolic and diastolic blood pressure (Table S1), and transiently increased plasma noradrenaline (Figure 1G) but not adrenaline concentrations (Figure S2). Cold exposure slightly decreased arterial glucose but did not increase clearance of 6,6-[^2^H]_2_-labeled glucose (Figures 1E and 1F). Cold exposure increased whole-body lipolysis as demonstrated by arterial non-esterified fatty acids and the rate of appearance of glycerol (Figures 1I and 1J). Cold exposure did not alter arterial lactate or pyruvate levels but transiently decreased glutamate concentrations (Figure S2).

#### Adipose Tissue Measurements

##### Warm Conditions

**Figure 2:**
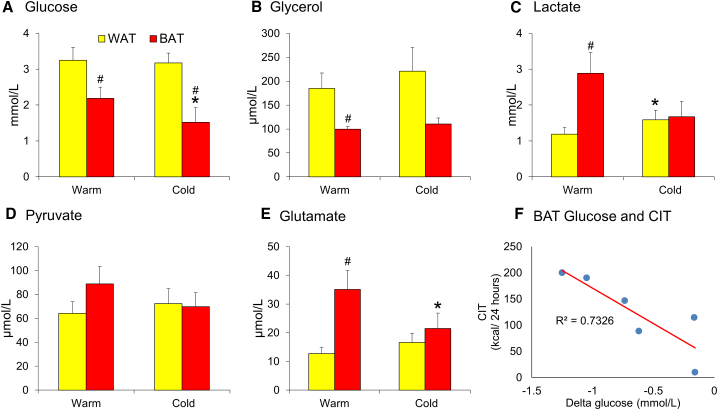
WAT and BAT Dialysate Concentrations Data are mean ± SEM for n = 6 subjects for dialysate concentrations measured using a flow rate of 0.3 μL/min. (A and B) Glucose (A) and glycerol (B) concentrations were lower in BAT (red columns) than in WAT (yellow columns) during warm conditions. (C–E) Lactate (C) and glutamate (E) concentrations were higher in BAT than WAT during warm conditions, while (D) pyruvate concentrations were similar between depots. Cold exposure reduced (A) glucose and (E) glutamate concentrations in BAT and (C) increased lactate concentrations in WAT. (F) The reduction in BAT glucose concentration during cold exposure strongly correlated with cold-induced thermogenesis (CIT). Data were analyzed by repeated measures ANOVA with *post hoc* LSD testing or using the Pearson correlation coefficient. ^∗^p < 0.05 versus warm conditions; ^#^p < 0.05 versus WAT.

Glucose concentrations in BAT were lower compared with WAT (Figure 2A), indicating substantial glucose uptake by BAT in warm conditions not typically associated with thermogenesis. Glycerol concentrations were lower in BAT than in WAT (Figure 2B), consistent with either reduced lipolysis or enhanced glycerol recycling in BAT. Lactate concentrations were higher in BAT, in keeping with increased glycolysis (Figure 2C), while pyruvate levels were similar in both tissues (Figure 2D). In addition, glutamate concentrations were higher in BAT, potentially due to reduced uptake (Figure 2E).

##### Cold Conditions

Interstitial BAT glucose levels decreased further during cold exposure, consistent with previous PET data identifying substantial cold-induced glucose uptake by BAT (Figure 2A). Similarly, cold decreased glutamate concentrations only in BAT (Figure 2E). Cold increased lactate concentrations in WAT (Figure 2C) but did not alter glycerol or pyruvate concentrations in WAT or BAT (Figures 2B and 2D). The reduction in BAT glucose concentrations closely correlated with cold-induced thermogenesis (Figure 2F, p < 0.01).

#### Quantifying Net Substrate Balance across BAT

**Figure 3:**
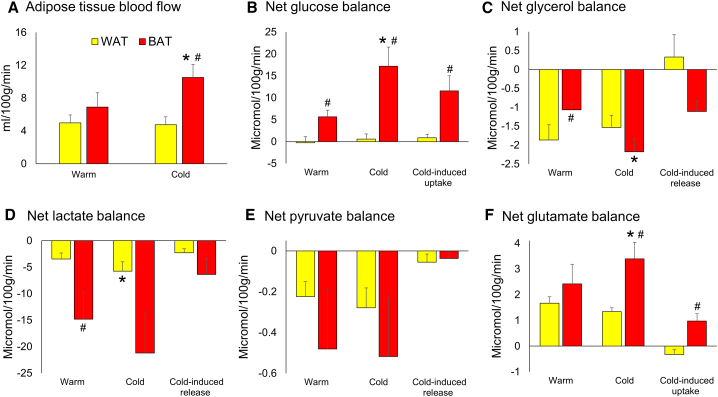
Net Substrate Balance in WAT and BAT (A–F) Data are mean ± SEM for (A) n = 6 and (B–F) n = 5 subjects. (A) Cold exposure increased blood flow in BAT (red columns) but not in WAT (yellow columns). There was (B) increased net glucose uptake, (C) decreased glycerol release, and (D) increased lactate release in BAT than WAT during warm conditions. Cold exposure increased (B) net glucose uptake, (C) glycerol release, and (F) glutamate uptake by BAT. Cold exposure increased (D) net lactate release by WAT. Data were analyzed by repeated measures ANOVA with *post hoc* LSD testing. ^∗^p < 0.05 versus warm conditions; ^#^p < 0.05 versus WAT.

The use of ^133^xenon to measure adipose tissue blood flow in “real time” allowed quantification of net balance of the above measurements. While blood flow was similar between WAT and BAT depots during warm conditions, cold exposure substantially increased blood flow only in BAT as seen previously (Figure 3A) (Astrup et al., 1985, Orava et al., 2011). The increase in BAT blood flow did not correlate with cold-induced thermogenesis (R^2^ = 0.057). The arterial and interstitial measurements were used to quantify net balance across WAT and BAT during warm and cold conditions, following calculation of the true interstitial concentrations at zero flow (Figure S3). There was detectable glucose uptake only by BAT during warm conditions (p < 0.05 versus 0) (Figure 3B). Cold exposure increased net glucose uptake by BAT by 11.5 ± 3.5 μmol/100 g tissue/min (p < 0.05) but not by WAT. There was net uptake of glutamate by WAT and BAT during warm conditions (both p < 0.05 versus 0) (Figure 3F), but cold only increased glutamate uptake by BAT. There was higher glycerol release by WAT than BAT during warm exposure; however, cold exposure only increased glycerol release by BAT (by 1.1 ± 0.3 μmol/100 g tissue/min) (Figure 3C). Net release of lactate was higher by BAT than WAT during warm conditions (Figure 3D), and cold increased lactate release by WAT during cold exposure. Pyruvate release by WAT and BAT was similar (both p < 0.05 versus 0) and unaltered by cold exposure (Figure 3E).

### Glycerol Recycling in Human BAT

**Figure 4:**
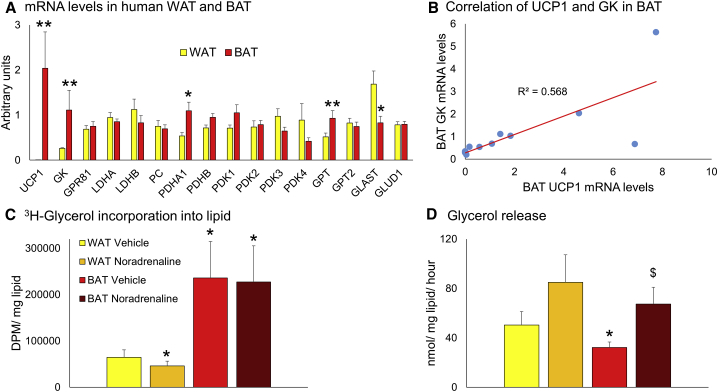
Glycerol Recycling and Release *In Vitro* (A) Data are mean ± SEM for n = 12 paired WAT (yellow columns) and BAT (red columns) samples obtained from patients undergoing elective surgery at room temperature. There were increased uncoupling protein 1 (UCP1), glycerol kinase (GK), alanine aminotransferase (GPT), and pyruvate dehydrogenase subunit A (PDHA), and decreased glutamate transporter (GLAST) mRNA levels in BAT compared with WAT. (B) GK and UCP1 mRNA levels strongly correlated in BAT (p < 0.01). (C and D) Data are mean ± SEM for n = 8 paired white and brown adipocytes treated with either vehicle or 10 μM noradrenaline (WAT, orange columns; BAT, brown columns) for (C) 6 or (D) 24 hr. (C) Incorporation of [^3^H]glycerol into cellular lipid following incubation (measured by disintegrations per minute [DPM]) was significantly increased in brown versus white adipocytes, correcting for cellular lipid content. (D) Glycerol release following incubation was reduced in vehicle-treated brown versus white adipocytes. Noradrenaline increased glycerol release by brown adipocytes and there was a similar trend (p = 0.07) in white adipocytes. Data were analyzed by (A) paired t test, (B) Pearson correlation coefficient, or (C and D) repeated measures ANOVA with *post hoc* LSD testing. ^∗^p < 0.05 ^∗∗^p < 0.01 versus WAT/WAT vehicle; ^$^p < 0.05 versus BAT vehicle.

The reduced glycerol release by human BAT during warm conditions could be secondary to reduced lipolysis or substantial glycerol recycling. In WAT, glycerol is not recycled due to the lack of the enzyme glycerol kinase; however, human peri-renal BAT has substantial glycerol kinase activity (Chakrabarty et al., 1983). To test this, we first measured glycerol kinase mRNA levels in paired human supraclavicular BAT and WAT samples (Ramage et al., 2016). Glycerol kinase expression was significantly higher in BAT than WAT (Figure 4A), and there was a strong correlation between glycerol kinase and uncoupling protein 1 mRNA levels (Figure 4B, p < 0.01). To test whether there was considerable glycerol recycling, [^3^H]glycerol incorporation into the lipid fraction was measured in primary human supraclavicular brown and white adipocytes cultured in the presence of either vehicle (to model thermoneutrality) or 10 μM noradrenaline (to model thermogenesis). Consistent with substantial glycerol recycling, ^3^H counts were increased severalfold in the brown compared with white adipocytes in both vehicle and noradrenaline-treated groups (Figure 4C). Noradrenaline did not alter ^3^H uptake in the brown adipocytes and decreased uptake in the white adipocytes. Consistent with the *in vivo* results, glycerol release by human brown adipocytes was reduced compared with white adipocytes in the vehicle-treated group and was increased by noradrenaline (Figure 4D). Similar results were obtained when [^3^H]glycerol uptake and glycerol release were normalized to cellular lipid (Figures 4C and 4D) or protein content (Figure S4).

### Other Intermediates in Human BAT

The high lactate concentrations in BAT may have an important physiological role. Lactate inhibits lipolysis through the GPR81 receptor, which is highly expressed in human BAT (Liu et al., 2009, Ahmed et al., 2010). Supporting this, we found high expression of GPR81 in both human WAT and BAT samples (Figure 4A), suggesting that the high BAT lactate concentrations observed during warm conditions may inhibit lipolysis and ensure that BAT triglyceride stores are rapidly replenished and maintained for subsequent hydrolysis during cold-induced thermogenesis. *LDHA* and *LDHB* mRNA levels were similar between WAT and BAT (Figure 4A).

There were increased mRNA levels of pyruvate dehydrogenase (PDH) subunit A (and a trend for increased *PDHB* [p = 0.06]) in BAT compared with WAT while levels of the PDH kinases were similar in both depots (Figure 4A), suggesting that pyruvate may be used as a substrate for the tricarboxylic acid (TCA) cycle in human BAT. We found low mRNA levels of the glutamate transporter GLAST in BAT compared with WAT (Figure 4A), suggesting that the high interstitial BAT glutamate levels in the warm may be due to reduced uptake. In addition, mRNA levels of the enzyme alanine aminotransferase (*GPT*), which converts alanine and α-ketoglutarate to pyruvate and glutamate, respectively, were increased in human BAT (Figure 4A), which could indicate enhanced glutamate synthesis and release by BAT. Glutamate dehydrogenase and *GPT2* mRNA levels were similar between WAT and BAT.

## Discussion

We adapted microdialysis as a novel tool to examine human BAT physiology *in vivo* and quantified uptake of substrates and release of intermediates by measuring arterio-interstitial differences in combination with real-time measurement of adipose tissue blood flow using ^133^xenon. We discovered that human BAT is metabolically active even during warm conditions not typically associated with BAT thermogenesis. There was glucose uptake by BAT but not WAT during warm conditions, while lactate release by BAT was higher than by WAT. Pyruvate release by BAT was also substantial, and taken together this suggests high rates of glycolysis in BAT during warm conditions. These data suggest that during warm conditions glucose may be used by BAT for glyceroneogenesis or fatty acid synthesis to increase triglyceride stores for subsequent hydrolysis when activated by cold. Glycerol release by BAT was reduced during warm conditions compared with WAT. Our *in vitro* data suggest that this is most likely due to glycerol recycling, although it is also possible that lipolysis is reduced, potentially due to elevated lactate levels inhibiting lipolysis through GPR81 (Liu et al., 2009). In addition, there was increased interstitial glutamate concentrations in BAT, potentially due to reduced uptake by the glutamate transporter. Glutamate inhibits glucose uptake by adipocytes (Nagao et al., 2017), so reducing intracellular glutamate may enhance glucose uptake by BAT compared with WAT.

Glucose was the primary substrate taken up by BAT during cold exposure; the increase in glucose uptake during cold activation (∼11.5 μmol/100 g/min) was similar to previous results obtained using ^18^FDG-PET/CT (Virtanen et al., 2009, Ouellet et al., 2012). The decrease in interstitial BAT glucose concentrations correlated with cold-induced thermogenesis, supporting previous PET/CT data (Yoneshiro et al., 2011, Chen et al., 2013). Lactate release was ∼21 μmol/100 g tissue/min during cold exposure and, although not statistically significant, increased by ∼6 μmol/100 g tissue/min compared with warm conditions. Our data strongly suggest that the majority of glucose uptake by BAT is not fully oxidized during BAT thermogenesis. Lactate production also accounts for a large proportion of glucose uptake by rodent BAT during thermogenesis (Ma and Foster, 1986). Two lactate molecules are produced from one glucose molecule so could account for all glucose uptake during warm conditions and over half (∼10.5 μmol/100 g tissue/min) of glucose uptake during BAT activation. Pyruvate production would only account for a small amount of glucose uptake (∼0.25 μmol/100 g tissue/min). If the remaining glucose uptake (∼6 μmol/100 g tissue/min would translate to ∼10 mmol glucose over a 24-hr period in our subjects) was fully oxidized, this would generate only ∼7 kcal/24 hr. This could still account for the majority of BAT thermogenesis according to some recent reports (Muzik et al., 2013, Din et al., 2016). However, the remaining glucose could be used for alternative processes such as glyceroneogenesis for triglyceride synthesis or alternatively for anaplerosis. The substantial glutamate uptake by BAT during cold activation may support the latter concept.

Recent data suggest that fatty acids hydrolyzed from local triglycerides are required for human BAT thermogenesis (Blondin et al., 2017). The glycerol release quantified by BAT during cold exposure (2.17 μmol/100 g tissue/min) would release ∼11 mmol of fatty acids in 24 hr. Assuming these are on average 16C fatty acids, if fully oxidized these could produce ∼25 kcal/24 hr. In addition, this calculation ignores the effect of glycerol recycling (which may be increased substantially during thermogenesis [Labbe et al., 2015]) and oxidation of circulating fatty acids taken up by BAT (Ouellet et al., 2012), so this may be a considerable underestimation. Our data suggest that fatty acids released from local triglycerides likely account for the majority of BAT thermogenesis. These estimates rely on several large assumptions, such as that substrate utilization remains constant during chronic activation and is similar in all BAT depots. In addition, more severe cold exposure may further increase substrate utilization by BAT. Surprisingly, we did not detect increased glycerol release by WAT during cold activation, despite increasing whole-body rate of appearance of glycerol. It is possible the microdialysis technique is not sensitive enough to detect the small local increase in WAT lipolysis. Conversely, abdominal adipose tissue may not contribute substantially to cold-induced lipolysis, although this is less likely. Recovery of fatty acids is unreliable using the microdialysis technique, so we were unable to measure fatty acid uptake or release by BAT (Jensen et al., 2007), although previous data suggest that fatty acid uptake is lower than glucose uptake by BAT (Ouellet et al., 2012).

There was substantial lactate and pyruvate release by BAT during warm and cold exposure. This did not increase during BAT activation, unlike in rodents (Ma and Foster, 1986); however, this may have occurred with more extreme and/or more prolonged cooling. We did find substantial uptake of glutamate by human BAT during cold activation. Pyruvate can be a substrate for BAT thermogenesis (Cannon and Nedergaard, 1979) and glutamate can be converted to α-ketoglutarate to enter the TCA cycle. The enzymes catalyzing those reactions were expressed in human BAT, and it is likely that BAT uses substrates other than fatty acids and glucose during thermogenesis. However, we did not measure protein or enzyme activity while mRNA levels of these enzymes may be regulated by cold exposure (Hao et al., 2015). Further work is required to determine the fate of these intermediates during BAT thermogenesis. The decrease in arterial blood glucose without increased glucose clearance may be due to reduced appearance of glucose. In support of this, the rate of appearance of glucose during cold was 8.0 ± 0.3 versus 8.5 ± 0.5 μmol/kg/min in the warm (p = 0.12), calculated using the non-steady-state Steele equation (Stimson et al., 2017).

The microdialysis technique offers several advantages over currently available methods to measure BAT activity *in vivo*. Measurement can be performed of multiple endogenous substrates in the tissue, is not reliant on exogenously administered tracers, can assess the effect of interventions in real time, and could be used to deliver drugs locally to determine the effect on BAT function. The disadvantages of microdialysis include the invasive nature of the technique and the radiation exposure from PET and CT scanning. The latter is required to ensure correct catheter placement as BAT depots are small (1–2 cm) and often surrounded by WAT. In addition, the slow microdialysis flow rates required to quantify tissue concentrations precludes measurement of very acute changes in BAT activation and small sample volumes are limiting. There are also potential confounders of the technique to consider. The extraction efficiency of all five analytes in dialysate was similar in WAT and BAT during warm and cold conditions, and so should reliably reflect the true tissue concentrations. Furthermore, the dialysate concentrations did not change over 3 hr of cold exposure, suggesting that equilibrium was achieved. A potential confounder could be the contribution of neighboring muscle to the interstitial BAT measurements, despite the short catheter membrane length and use of CT to ensure correct catheter placement. However, ^18^FDG uptake and O_2_ consumption by these muscles is much lower than BAT during cold exposure (Ouellet et al., 2012, Blondin et al., 2015, Din et al., 2016) and, if anything, would cause an underestimation of substrate utilization by BAT. In addition, the BAT lactate and glycerol concentrations and glucose uptake by BAT were substantially higher than previously reported in skeletal muscle (Orava et al., 2011, Muller et al., 1996, Rosdahl et al., 1998).

### Limitations of Study

There are some caveats with this protocol to consider. Firstly, due to the invasive nature of microdialysis we were only able to cannulate two BAT depots. We measured substrate utilization in one depot and it is possible that substrate utilization by other BAT depots may differ. Therefore, our estimations of substrate utilization by all BAT should be interpreted with caution. Secondly, in our protocol, subjects were exposed to mild cold for only 3 hr. It is possible that more severe or more prolonged exposure would have altered substrate utilization by BAT substantially, as this may deplete local triglyceride stores. Finally, we only studied young lean men so are unable to determine how age, weight, or sex alters substrate utilization by BAT.

To conclude, we developed microdialysis as a novel technique to measure *in vivo* BAT activity in humans. We determined that there is substantial metabolic activity in BAT even during warm conditions with increased glucose uptake, lactate production, and reduced glycerol release than WAT. Cold-induced BAT activation substantially increases glucose uptake and glycerol release from lipolysis of BAT triglyceride stores, and most likely leads to utilization of additional substrates such as glutamate. Improved understanding of substrate utilization by BAT in humans may identify new targets to activate this tissue to improve metabolic health.

## STAR★Methods

### Key Resources Table

**Table:** 

REAGENT or RESOURCE	SOURCE	IDENTIFIER
**Biological Samples**		

Human blood and dialysate samples	Healthy male volunteers	N/A
Human white and brown adipose tissue	Edinburgh Adipose Tissue Biobank	N/A

**Chemicals, Peptides, and Recombinant Proteins**		

^18^Fluourodeoxyglucose	Edinburgh Clinical Research Imaging Centre	N/A
6,6-[^2^H]_2_-Glucose	Euroiso-top	IND570P
1,1,2,3,3-[^2^H]_5_-Glycerol	Euroiso-top	IND575P
^133^Xenon	IDB Holland	N/A
2-[^3^H]-Glycerol	PerkinElmer	NET022L001MC

**Experimental Models: Cell Lines**		

Primary human white and brown adipocytes	Edinburgh Adipose Tissue Biobank	N/A

**Oligonucleotides**		

See Table S3	This paper	N/A

**Software and Algorithms**		

SPSS version 22	IBM	N/A
GMS411 Mediscint gamma counter system	John Caunt Scientific Ltd	http://www.johncaunt.com/products/gms411/

### Contact for Reagent and Resource Sharing

Further information and requests for resources and reagents should be directed to and will be fulfilled by the Lead Contact, Roland Stimson (roland.stimson@ed.ac.uk).

### Experimental Model and Subject Details

#### In Vivo Study Participants

Six healthy lean men (subject information in Table S1) were recruited using the following inclusion criteria: aged 18-35 years; body mass index (BMI) 18.5-25 kg/m^2^; weight change <5% in last 6 months; no acute or chronic medical conditions; on no regular medications; alcohol intake ≤ 21 units/ week; no claustrophobia; no allergy to local anaesthetic. Approval was obtained from the South East Scotland Research Ethics Committee and informed consent was obtained from each subject.

#### In Vitro Study Participants and Cell Culture

White and brown adipose tissue samples were obtained from euthyroid subjects undergoing elective thyroid or parathyroid surgery (subject details in Table S2) in the Royal Infirmary of Edinburgh as previously described (Ramage et al., 2016). Approval was obtained from the South East Scotland Research Ethics Committee and informed consent was obtained from each subject. During their operation, a small quantity of BAT was obtained from the central compartment of the neck superior to the clavicle and deep to the lateral thyroid lobe either adjacent to the longus colli muscle or to the oesophagus. Paired WAT samples were obtained from the superficial subcutaneous neck. Tissue was either immediately frozen at −80°C for subsequent qPCR (n = 12, 11 female/1 male) or the stromal vascular fraction isolated for culture (n = 8, 6 female/2 male) as previously described (Ramage et al., 2016). Adipose tissue for isolation was cut into small pieces and digested using 0.2% collagenase type 1 in Krebs-Heinseleit buffer at 37°C for 45 min. Following centrifugation at 600 *g* for 10 min at 20°C, the re-suspended pellet was passed through a 100 μm filter and subjected to centrifugation at 200 *g* for 5 min at 20°C. The pellet was re-suspended in DMEM containing 10% fetal bovine serum (FBS) and 1 nM human fibroblast growth factor-basic (Peprotech, Rocky Jill, New Jersey) and cultured in 6-well plates at 37°C. Medium was changed every 2-3 days and cells were passaged at 80% confluence. Confluent cells were differentiated in DMEM containing 10% FBS with the addition of 1 nM tri-iodothyronine, 20 nM insulin, 500 μM IBMX, 500 nM dexamethasone and 125 μM indomethacin for 5 days (days 2-7). Thereafter, cells were cultured in DMEM containing 10% FBS, 1 nM tri-iodothyronine and 20 nM insulin until the experiments which were performed between days 14–16.

### Method Details

#### In Vivo Study Protocol

Subjects attended the Edinburgh Clinical Research Facility after overnight fast wearing the same clothes (clo thickness ∼0.5-0.6) on 2 consecutive days and avoided alcohol and exercise for 48 hr prior to each visit. On study visit 1 (‘BAT finding visit’), measurements were performed of height, weight, and body fat using bioimpedance (using an Omron BF-302). A cannula was placed in an antecubital vein and a CT needle guide (IZI Medial Products, Baltimore, MD) was placed posteriorly medial to the scapular area to aid the subsequent CT-guided insertions. Thereafter, subjects were placed in a room cooled to 17°C for 2 hr to activate BAT. After 1 hr of cold exposure, subjects received an intravenous injection of 185 MBq ^18^F-fluorodeoxyglucose (^18^FDG). Subjects were asked and assessed clinically for shivering every 15 min; no shivering was detected clinically or noted by any subject during either visit. PET/CT was then performed as previously described (Ramage et al., 2016) with the exception that subjects lay prone in the scanner while the scans were performed using 5-min beds. The PET/CT scan was reviewed to ensure there was enough ^18^FDG uptake by BAT to allow insertion of the microdialysis catheter and ^133^Xenon at visit 2. Subjects were allowed home and kept the needle guides in place overnight.

Upon arrival for study visit 2 (microdialysis visit), anthropometric measurements were performed as per visit 1. A cannula was placed in an ante-cubital fossa vein and baseline bloods obtained. Intravenous infusions of 6,6-[^2^H]_2_-glucose (at a rate of 0.22 μmol/kg/min following a 17.6 μmol/kg bolus) and 1,1,2,3,3-[^2^H]_5_-glycerol (at 0.11 μmol/kg/min following a 1.6 μmol/kg bolus) were commenced at T= –120 min for 8 hr (Figure S1). Subjects were moved to the CT suite at the Royal Infirmary of Edinburgh and lay prone on a 64-multidetector row CT scanner (Aquilion 64, Toshiba Medical Systems, Crawley, United Kingdom). A microdialysis catheter (63 microdialysis catheter (length 60 mm, membrane 10 mm), M Dialysis, Johanneshov, Sweden) was inserted into the supraclavicular BAT under local anaesthetic (1% lidocaine), using the PET/CT images from study visit 1 under initially ultrasound (using a LOGIQ S7 Expert, GE Medical Systems Ltd, Buckinghamshire, United Kingdom) and then CT-guidance to ensure the CT-visible catheter tip was correctly positioned in the PET positive BAT (Figure S1). ^133^Xenon (2 MBq) was then injected into the contralateral supraclavicular BAT depot under CT-guidance using a 22G spinal needle to measure adipose tissue blood flow continuously throughout the study protocol (Goossens and Karpe, 2008). Subjects returned to the Clinical Research Facility and were placed in a room at ∼25°C (warm room). A second microdialysis catheter was placed in subcutaneous abdominal white adipose tissue (WAT) ∼5 cm lateral to the umbilicus and 2MBq ^133^Xenon was injected in the contralateral WAT at the same depth as the catheter. An arterial catheter (Leadercath, Vygon Ltd, Swindon, United Kingdom) was inserted in a radial artery for blood sampling and measurement of blood pressure. At t = 0 min, microdialysis was commenced in both WAT and BAT depots using M Dialysis 107 pumps at a flow rate of 0.3 μL/min. Blood flow was measured continuously using a gamma counter (John Caunt Scientific, Lancashire, United Kingdom). Energy expenditure was measured each hour by indirect calorimetry. Arterial samples and dialysate were collected regularly (Figure S1). Skin temperature was measured regularly from the left and right distal forearm using skin temperature probes (YSI 409 series, Henleys Medical Supplies Ltd, Hertfordshire, United Kingdom) with the results presented as the mean of the two sides. At t = 180 min, subjects were moved to a room cooled to ∼17°C (cold room). Identical measurements were performed during cold exposure.

#### PET/CT Protocol and Analysis

All subjects lay prone in a hybrid PET/CT scanner (Biograph mCT, Siemens Medical Systems, Erlangen, Germany). Subjects underwent an initial low dose CT for attenuation correction (non-enhanced, 120 kV) with tube current modulation applied 20 mAs quality reference followed by static PET imaging of the upper body using 5 min beds. Images were analysed using PMOD version 3.409 (PMOD technologies, Zurich, Switzerland). ^18^FDG uptake by BAT was quantified by measuring the mean uptake in KBq from all pixels with uptake >2KBq/cc which corresponded to tissues with a radio-density on the CT scan with Hounsfield unit (HU) values within the expected range for adipose tissue (from −150 to −30 HU). The total ^18^FDG uptake by BAT was calculated as the mean uptake in KBq/cc multiplied by the volume of active BAT in cc.

#### Indirect Calorimetry

Energy expenditure was measured for 15 min each hour using a ventilated-hood indirect calorimeter (GEM Nutrition, Cheshire, United Kingdom). The first 5 min of data were discarded and the mean value for the final 10 min recorded each hour. Energy expenditure (EE) is presented as the mean of three values obtained during warm and cold exposure. Cold-induced thermogenesis was calculated by subtracting the mean EE in the warm room from the EE in the cold room.

#### Microdialysis Technique

##### Microdialysis Infusion Method Development

Initial work was performed testing flow rates of 0.1, 0.2, 0.3, 0.5, 1.0, 2.0 and 5.0 μL/min using the 63 M Dialysis catheters *in vitro* to determine whether equilibrium was achieved. Microdialysis was performed in solutions containing 3 mmol/L glucose, 200 μmol/L glycerol, 1.5 mmol/L lactate, 70 μmol/L pyruvate, and 20 μmol/L glutamate at the above rates (n=4). The mean dialysate concentrations of all 5 compounds of interest were within 7% of the known concentrations when using flow rates of 0.1, 0.2, 0.3 and 0.5 μL/min, and when using a flow rate of 1.0 μL/min for lactate, pyruvate, and glycerol. Concentrations decreased substantially at the higher flow rates.

To determine the extraction efficiency of these compounds in white and brown adipose tissue *in vivo*, 4 healthy male subjects (aged 26.8 ± 3.0 years, BMI 22.5 ± 0.7 kg/m^2^) were recruited. Local ethical approval and informed consent was obtained from all subjects. The visit 1 protocol was identical to that described above, which identified substantial ^18^FDG uptake by BAT in all subjects. Subjects returned after overnight fast the following morning during which a 63 M Dialysis catheter was placed in the supraclavicular BAT under CT guidance and in subcutaneous abdominal WAT. Two of the subjects were then kept in a room at 21°C and the other two subjects were kept at 17°C for the duration of the visit to determine the extraction efficiency during both warm and cold conditions. T1 perfusion fluid was infused at 0.1, 0.2, 0.3, 0.5, 1.0, 2.0 and 5.0 μL/min and dialysate was collected regularly throughout. Unlike *in vitro*, mean concentrations of glucose, glycerol, lactate, pyruvate, and glutamate did not reach steady state even at the lower flow rates. The tissue concentration of each compound at zero flow was determined using the perfusion rate method (Stahle et al., 1991) in order to quantify arterio-interstitial differences. The flow rate was plotted against 1/mean concentration, which had linear R^2^ values of >0.96 for all 5 compounds of interest when the 5.0 μL/min flow rate was excluded (Figure S3). At 0.3 μL/min (the flow rate chosen for the main study protocol), the calculated recovery of the various compounds in the warm was 69% (WAT) and 72% (BAT) for glucose, 84% (both WAT and BAT) for lactate, 74% (WAT) and 76% (BAT) for pyruvate, 33% (WAT) and 35% (BAT) for glycerol and 80% (both WAT and BAT) for glutamate. The calculated recovery during cold conditions was 72% (WAT) and 74% (BAT) for glucose, 82% (WAT) and 78% (BAT) for lactate, 74% (WAT) and 73% (BAT) for pyruvate, 33% (WAT) and 32% (BAT) for glycerol and 82% (WAT) and 88% (BAT) for glutamate.

##### Microdialysis Study Protocol

Following insertion of a 63 M Dialysis catheter in the supraclavicular BAT and abdominal WAT T1 perfusion fluid was infused through both M Dialysis 107 pumps (M Dialysis, Johanneshov, Sweden) at a rate of 0.3 μL/min. Dialysate was collected in microvials each hour from the WAT and BAT catheters respectively and stored in microvial racks at −80°C until analysis. Dialysate collected during the first hour of infusion (from T=0 to T+60 min) was discarded due to tissue trauma from the insertions potentially confounding results. Three of the subjects had all 6 dialysate samples collected as per study protocol. In these subjects dialysate concentrations of glucose, glycerol, pyruvate, lactate and glycerol did not change during the same conditions (e.g. results from t+120 and t+180 were similar as were concentrations at t+240, t+300 and t+360 min). However, in three subjects there were temporary blockages in one of the microdialysis catheters leading to loss of one or more samples although all subjects had at least one dialysate sample collected during both warm and cold conditions. Therefore, the data are presented as the mean of all results obtained from each subject in the warm and cold conditions.

#### In Vitro Study Protocol

Paired primary human brown and white adipocytes were incubated in a Krebs-Ringer bicarbonate phosphate buffer solution with 25 mmol/L glucose at pH 7.4 with 10μM noradrenaline or vehicle for either 6 or 24 hr depending on the experiment. Glycerol release by the adipocytes was measured following 24 hr incubation as previously described (Stimson et al., 2017). To measure incorporation of ^3^H-glycerol into lipid (a measure of glycerol recycling), 100 μM glycerol and 6.25 μM 2-[^3^H]-glycerol (PerkinElmer, Waltham, MA) was added to the above medium in a subset of adipocytes for 6 hr by adapting a previously published protocol (Kiskinis et al., 2014).

##### ^3^H-Glycerol Incorporation Assay

The incorporation of ^3^H-glycerol into lipid was measured in triplicate by adapting a previously published protocol (Kiskinis et al., 2014). Cells were serum starved for 3 hr and then incubated as above. Following the 6-hour incubation, cells were washed three times in 4°C PBS then methanol (900 μL) was added to wells, which were then scraped on ice to lyse the cells prior to transfer to a new tube. Chloroform (450 μL) and then water (450 μL) were added to the mixture, vortexed, and then subjected to centrifugation at 3,200 *g* for 20 min at room temperature. The infranatant (organic lipid layer) was transferred to a new tube, dried down under N_2_ at 50°C prior to reconstitution in scintillation fluid (Ultima Gold, PerkinElmer, Waltham, MA) and quantification by liquid scintillation counting. To account for variability in the differentiation of the adipocytes, results were normalised either to the cellular lipid content as measured by Oil Red O or to cellular protein content.

#### Biochemical Assays

Serum insulin (DRG Instruments, Marburg, Germany), plasma adrenaline and noradrenaline concentrations (LDN, Nordhorn, Germany) were measured by ELISA. NEFAs were measured using a colorimetric assay (Wako Diagnostics, Richmond, Virginia, USA). Serum glutamate (Abcam, Cambridge, United Kingdom), lactate and pyruvate (Cayman Chemical Company, Ann Arbor, MI) were measured using fluorimetric kits. Glycerol in the culture medium was measured using a colourimetric kit (Sigma, Poole, UK) and protein in cell lysates was measured in duplicate using the DC protein assay (Bio-Rad, Hercules, CA) as previously described (Stimson et al., 2017). Cellular lipid content was measured using Oil Red O (Sigma, Poole, UK) as previously described (Janderova et al., 2003).

Plasma glucose, 6,6-[^2^H]_2_-glucose (D2-glucose), glycerol and 1,1,2,3,3-[^2^H]_5_-glycerol (D5-glycerol) were measured using gas chromatography mass spectrometry as previously described (Macfarlane et al., 2014). Dialysate glucose, glycerol, lactate, pyruvate, and glutamate concentrations were measured using an ISCUS clinical microdialysis analyser (M Dialysis, Johanneshov, Sweden).

#### Quantitative Real-Time PCR in Human Brown and White Adipose Tissue

qPCR was performed as previously described (Ramage et al., 2016). mRNA levels are presented as the ratio of genes of interest to the mean of internal control genes (18S and cyclophilin A). Primer sequences and probes numbers are detailed in Table S3.

#### Tracer Kinetics and Net Balance Equations

The rate of appearance (Ra) of glycerol was calculated using the following equation:

Ra glycerol = D5-Glycerol infusion rate/(D5-Glycerol/Glycerol tracer: tracee ratio)

The metabolic clearance rate (MCR) of glucose was calculated using the following equation:

MCR glucose = D2-Glucose infusion rate/D2-Glucose concentration

The rate of disposal of glucose was also calculated using the above equation for Ra glycerol by adapting the respective tracer and tracee values. Both the values for MCR glucose and Rd glucose were unchanged by cold exposure, the results presented are for the MCR glucose.

Net balance of glucose was calculated using the following equation, which was adapted by insertion of relevant values to calculate net balance of the other compounds of interest:

Net glucose balance = ([Glucose_Artery_] – [Glucose_Interstitial_]) x blood flow

The interstitial concentrations used in the above equation were the calculated tissue concentrations at zero flow. This was calculated for each compound by dividing the concentration measured in the dialysate by the extraction efficiency value at 0.3 μL/min (e.g., for WAT glucose during warm conditions the measured concentration was divided by 0.69).

#### Measurement of Adipose Tissue Blood Flow Using ^133^Xenon

Adipose tissue blood flow was measured as previously described (Goossens and Karpe, 2008) using a gamma counter (John Caunt Scientific Ltd, Bury, United Kingdom) with measurements performed every minute. The tissue-blood partition coefficient of ^133^Xe for adipose tissue (λ) was calculated using the following equation (Goossens and Karpe, 2008):

λ (ml/g) = [V(S_L_/S_P_ − 1) + 1] / [Hct(S_C_/S_P_ − 1) + 1]

In this equation V is the lipid fraction of adipose tissue, S_L_ (solubility in lipid) is 1.8276 mL/g, S_P_ (solubility in plasma) is 0.0939 mL/mL, Hct is the hematocrit (expressed as a fraction), and S_C_ (solubility in red blood cells) is 0.2710 mL/mL. It was not possible to measure V in this group of participants, as such 0.72 was used for human supraclavicular BAT as has been determined previously using MRS in lean individuals, while 0.87 was used for WAT (Raiko et al., 2015). The mean partition coefficients for these subjects were 9.4 ± 0.1 (WAT) and 7.9 ± 0.1 (BAT).

Adipose tissue blood flow (ATBF) was calculated using the following equation:

ATBF (mL/100g tissue/min) = slope of semilog plot (ln counts/s) × λ (mL/g) × 100 (g) × 60 (s)

### Quantification and Statistical Analysis

Data are presented as mean ± SEM. Data were tested for normal distribution using the one-sample Kolmogorov-Smirnov test and were log transformed if not normally distributed. Comparisons between two related groups (e.g. energy expenditure during warm and cold conditions) were examined using the paired t test, while comparisons involving three or more related groups were analysed using repeated measures ANOVA with post hoc testing using Fisher’s least significant difference (LSD) test. The specific statistical tests used are detailed in the respective figure legends. Associations were tested using Pearson’s correlation coefficient. p < 0.05 was considered significant. SPSS version 22 was used for all analyses.

## Supplemental Information

Document S1. Figures S1–S4 and Tables S1–S3

Document S2. Article plus Supplemental Information
