# Supplementary material for: Substantial Metabolic Activity of Human Brown Adipose Tissue during Warm Conditions and Cold-Induced Lipolysis of Local Triglycerides
Source: Cell Metab. 2018 Jun 5;27(6):1348–1355.e4. doi: 10.1016/j.cmet.2018.04.020 (PMC5988566; doi:10.1016/j.cmet.2018.04.020)
Supplement: Document S1. Figures S1–S4 and Tables S1–S3 [file mmc1.pdf]

Cell Metabolism, Volume *27*

Supplemental Information

Substantial Metabolic Activity of Human Brown Adipose Tissue during Warm Conditions and Cold-Induced Lipolysis of Local Triglycerides

Graeme Weir, Lynne E. Ramage, Murat Akyol, Jonathan K. Rhodes, Catriona J. Kyle, Alison M. Fletcher, Thomas H. Craven, Sonia J. Wakelin, Amanda J. Drake, Maria-Lena Gregoriades, Ceri Ashton, Nick Weir, Edwin J.R. van Beek, Fredrik Karpe, Brian R. Walker, and Roland H. Stimson

Supplemental Information

Figure S1 (related to experimental procedures) **In vivo *study protocol***

Study protocol for A) visit 1 and B) visit 2. A) Subjects were placed in a room cooled to 17°C at t = 0 minutes. At t+60 minutes an intravenous injection of 185MBq ^{18}fluorodeoxyglucose (^{18}FDG) was administered and subjects remained in the cold room until a PET/CT scan was performed 1 hour later. B) At study visit 2 intravenous infusions of 6,6-[^{2}H]$_{2}$-glucose and 1,1,2,3,3-[^{2}H]$_{5}$-glycerol were commenced at t=-120 minutes. Microdialysis (MD) catheters and ^{133}Xenon (^{133}Xe) were placed in supraclavicular BAT and abdominal subcutaneous WAT and an arterial catheter (A-line) inserted in the radial artery. Subjects were placed in a warm room (24-25°C) from t=0 minutes for 3 hours then were transferred to a cold room (17°C) for a further 3 hours. Arterial blood (A) and dialysate (D) samples were obtained regularly with indirect calorimetry (IC) performed hourly. C) A CT image from a subject showing the presence of ^{133}Xe gas and the gold tipped MD catheter positioned in BAT depots.

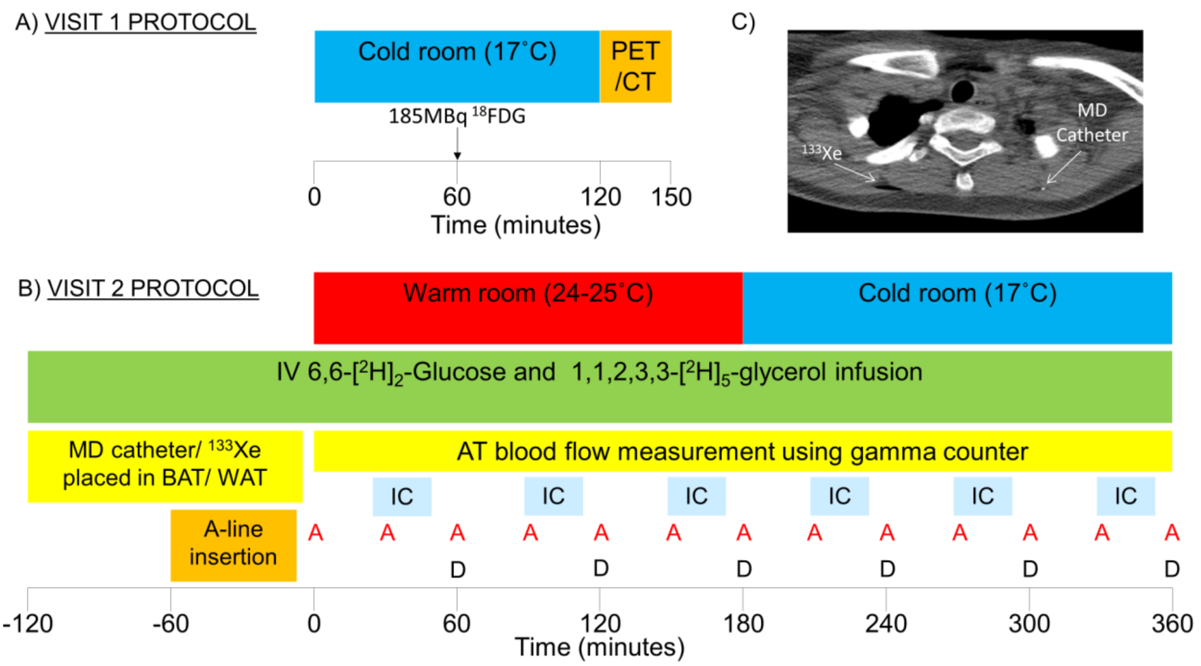

Figure S2 (related to Figure 1) Arterial measurements

*Data are mean ± SEM from n=5 subjects for A) lactate, B) pyruvate, C) glutamate and D) adrenaline concentrations during warm and cold exposure. Cold did not alter lactate, pyruvate or adrenaline concentrations but transiently decreased glutamate concentrations. Data were analysed by repeated measures ANOVA with post hoc LSD testing. *P<0.05 vs warm conditions.*

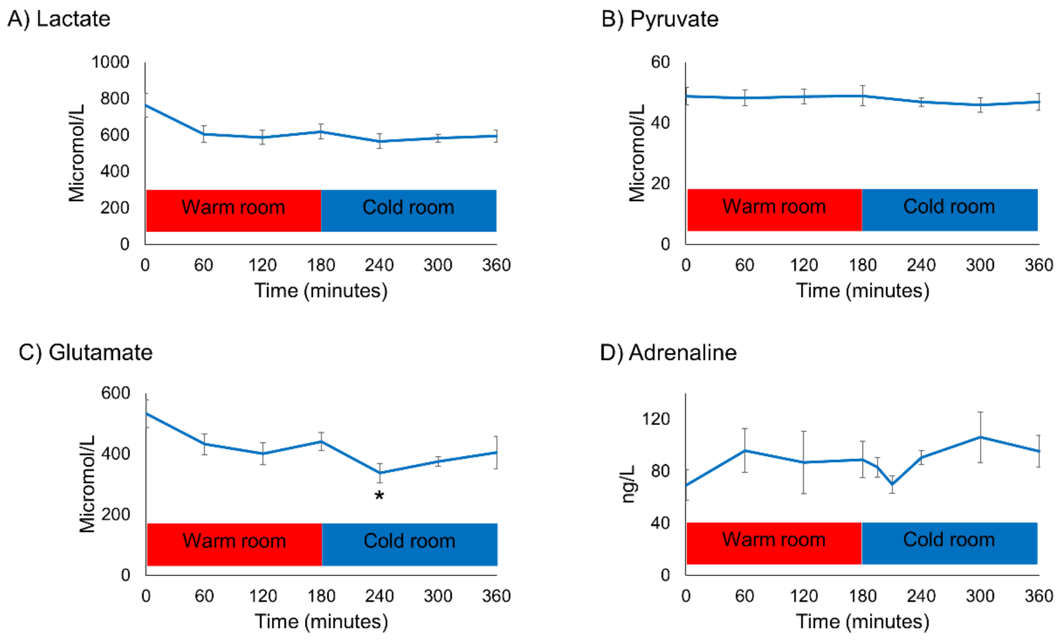

Figure S3 (related to experimental procedures) Microdialysis extraction efficiencies during warm and cold exposure

Data are mean values from four healthy men placed in either A-E) warm (~21°C, n=2) or F-J) cold (~17°C, n=2) conditions. Dialysate samples were collected from WAT (yellow circles) and BAT (red circles) during different flow rates of the microdialysis pump for measurement of A,F) glucose, B,G) glycerol, C,H) lactate, D,I) pyruvate, and E,J) glutamate. Linear correlations were observed when the flow rates were plotted against 1/concentration of all 5 compounds of interest. These equations were used to calculate the true tissue concentrations at zero flow.

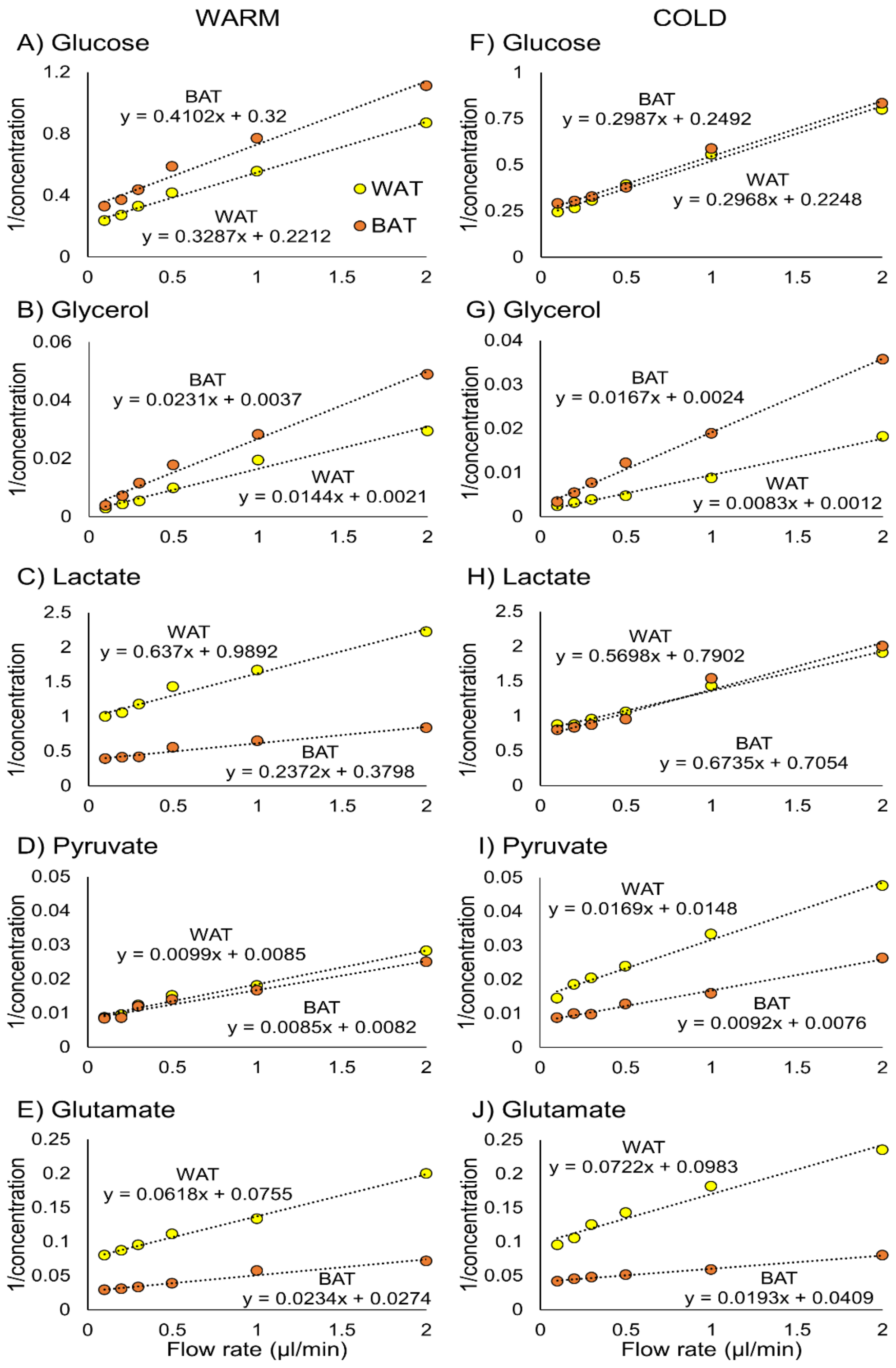

Figure S4 (related to Figure 4) Glycerol recycling and release **in vitro**

*Data are mean ± SEM for n=8 paired vehicle treated white (yellow columns) and brown (red columns) and 10μM noradrenaline treated white (orange columns) and brown (brown columns) adipocytes. Lipid content was measured by Oil red O following A) 6 and B) 24 hours treatment. C) Total cellular protein following 24 hours treatment. Lipid and protein content was reduced in the brown adipocytes consistent with reduced differentiation, hence results for ^{3}H-glycerol uptake and glycerol release were corrected for lipid and protein content which did not alter the results. D) Incorporation of ^{3}H-glycerol into lipid following 6 hours incubation as measured by disintegrations per minute (DPM) was significantly increased in brown vs white adipocytes when corrected for total cellular protein, consistent with substantial glycerol recycling. E) Glycerol release following 24 hours incubation was reduced in vehicle treated brown vs white adipocytes when corrected for total cellular protein, while noradrenaline substantially increased glycerol release in both brown and white adipocytes. Data were analysed by repeated measures ANOVA with post hoc LSD testing. *P<0.05 vs WAT vehicle; $ P<0.05 vs BAT vehicle.*

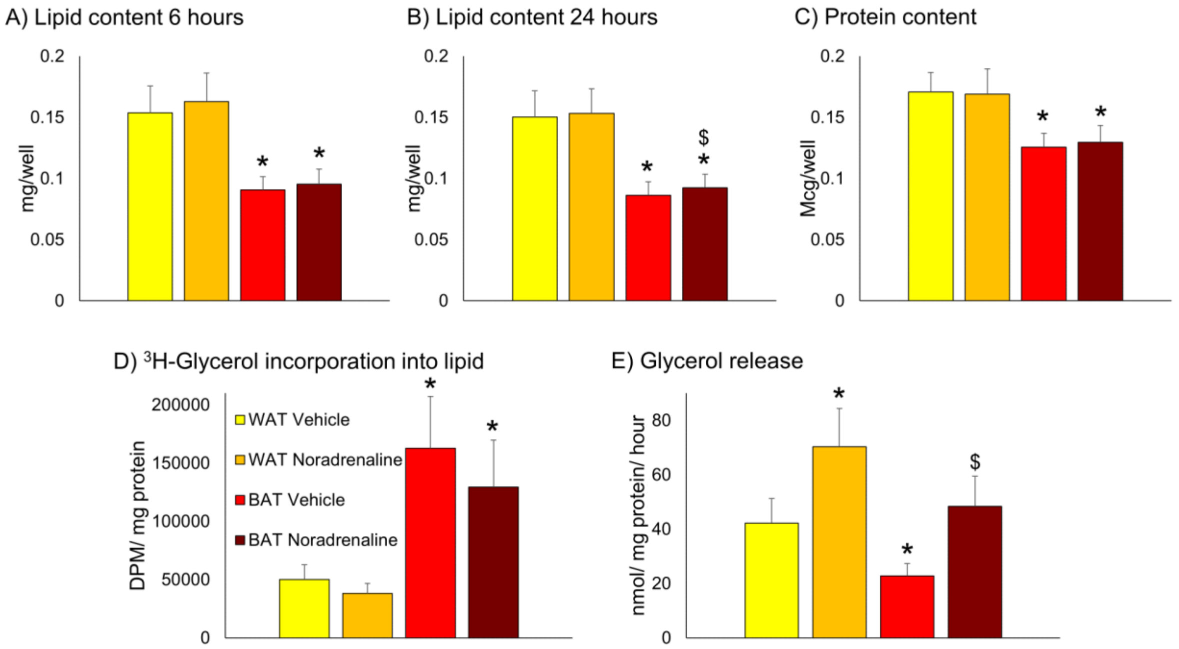

Table S1 (related to experimental model and subject details) Anthropometric and fasting biochemical data from* in vivo *study

Data are mean ± SEM for measurements in warm conditions and during cold exposure. Data were analysed by paired t tests. $^*P<0.05$, $^{***}P<0.001$ *vs measurements in warm room.*

**Number of subjects**	6	
**Age (years)**	21.3 ± 0.8	
**Body mass index (kg/m^2)**	22.7 ± 0.6	
**Fat mass (kg)**	14.2 ± 2.4	
**Fasting glucose (mmol/L)**	4.9 ± 0.1	
**Fasting insulin (mU/L)**	6.6 ± 1.0	
	**Warm room**	**Cold room**
**Room temperature (°C)**	24.7 ± 0.4	16.9 ± 0.2***
**Peripheral skin temperature (°C)**	32.2 ± 0.5	26.5 ± 0.6***
**Systolic blood pressure (mmHg)**	131 ± 2	138 ± 2***
**Diastolic blood pressure (mmHg)**	70 ± 2	74 ± 4*
**Heart rate (bpm)**	67 ± 2	66 ± 3

Table S2 (related to experimental model and subject details) Subject characteristics for **in vitro** *studies*

**Subject**	**Operation**	**Underlying diagnosis**	**Age (years)**	**Sex**	**Body mass index (kg/m^2)**
**Whole tissue**					
**1**	Parathyroidectomy	Primary hyperparathyroidism	66	Female	28.3
**2**	Parathyroidectomy	Primary hyperparathyroidism	63	Female	33.3
**3**	Thyroid lobectomy	Papillary carcinoma of thyroid	45	Female	26.5
**4**	Thyroid lobectomy	Benign thyroid nodule	51	Female	30.7
**5**	Thyroid lobectomy	Follicular adenoma	48	Female	17.6
**6**	Thyroid lobectomy	Benign thyroid nodule	39	Female	27.7
**7**	Parathyroidectomy	Primary hyperparathyroidism	24	Male	27.7
**8**	Thyroidectomy	Graves' disease	27	Female	30.1
**9**	Thyroidectomy	Graves' disease	48	Female	32.3
**10**	Thyroid lobectomy	Benign thyroid nodule	27	Female	23.9
**11**	Thyroid lobectomy	Multinodular goitre	66	Female	24.5
**12**	Sub-total thyroidectomy	Graves' disease	36	Female	28.2
**Adipocyte cultures**					
**1**	Thyroidectomy	Multinodular goitre	40	Female	24.2
**2**	Thyroidectomy	Graves' disease	52	Female	24.9
**3**	Parathyroidectomy	Primary hyperparathyroidism	48	Male	25.6
**4**	Thyroidectomy	Graves' disease	42	Female	23.1
**5**	Parathyroidectomy	Primary hyperparathyroidism	55	Male	29.9
**6**	Thyroidectomy	Graves' disease	37	Female	23.0
**7**	Thyroidectomy	Graves' disease	38	Female	25.9
**8**	Thyroid lobectomy	Follicular adenoma	41	Female	26.5

Table S3 (related to experimental procedures) Primer sequences and probe numbers for quantitative real time PCR

Gene name	Primer sequences 5' to 3'	Roche UPL Probe number
RNA18S5 (18S)	F: cttccacaggaggcctacac	46
	R: cgcaaaatatgctggaact	
PPIA (cyclophilin A)	F: atgctggacccaacacaat	48
	R: tctttcactttgccaaacacc	
UCP1	F: ctcaccgcagggaaagaa	25
	R: ggttgcccaatgaatactgc	
GK	F: caatcttggaattataaagacctcaga	46
	R: cgaaaatgctgggacgaa	
HCAR1 (GPR81)	F: gggagcatcgtgttccttac	83
	R: ggggtggaccactttgaa	
PC	F: agcaagctcttcagcatgg	52
	R: aggggcactcatacaggaag	
GPT	F: gggaaggcacctaccacttc	66
	R: acttggcatggaacctgct	
GPT2	F: gatcctgcaggcttgtgg	64
	R: ttcacggatgcagttgacac	
GLUD1	F: tcaccatggagctagcaaaa	50
	R: acatctcccgctcacctgt	
PDHA1	F: gtccgagaggcaacaaggt	74
	R: aagtctgcagctccatcagg	
PDHB	F: aggaggctggccacagtt	17
	R: caggaaattgaacgcaggac	
PDK1	F: caagacctcgtgttgagacct	20
	R: acgtgatatgggcaatccat	
PDK2	F: gccaacatcatgaaagagatca	38
	R: aggaggctctggacatacca	
PDK3	F: tgtgtgaacagtattacctggtagc	11
	R: gtttgtctggcgctttgg	
PDK4	F: aggacgcgtttccaagttc	63
	R: tttatttgtctccccgcact	
LDHA	F: gtccttggggaacatggag	47
	R: ttcagagagacaccagcaaca	
LDHB	F: gatggattttgggggaacat	5
	R: aacacctgccacattcacac	
SLC1A3 (GLAST)	F: cggacaaattattacaatcagca	5
	R: attccagctgccccaatact	
